# Super-resolution Surface Microscopy of Conductors using Magnetic Resonance

**DOI:** 10.1038/s41598-017-05429-3

**Published:** 2017-07-14

**Authors:** Andrew J. Ilott, Alexej Jerschow

**Affiliations:** Department of Chemistry, New York University, 100 Washington Square East, New York, NY 10003 USA

## Abstract

The spatial resolution of traditional Magnetic Resonance Imaging (MRI) techniques is typically dictated by the strength of the applied magnetic field gradients, resulting in hard resolution limits of the order of 20–50 *μ*m in favorable circumstances. We demonstrate here a technique which is suitable for the interrogation of regions at specified distances below the surface of conducting objects with a resolution well below these limiting values. This approach does not rely on magnetic field gradients, but rather on the spatial variation of the radiofrequency field within a conductor. Samples of aluminium and lithium metal with different sizes and morphologies are examined with this technique using ^27^Al and ^7^Li NMR. In this implementation, the slice selectivity depends on the conductivity of the material, as well as on the frequency of operation, although in the most general case, the technique could also be used to provide spatial selectivity with arbitrary *B*_1_ field distributions in non-conductors.

## Introduction

Imaging using Magnetic Resonance (MR) techniques has typically relied on the ability to encode spatial information in the frequency or phase of the precessing nuclear spins^1, 2^. In MRI, this process is achieved through the application of magnetic field gradients, which has led to a plethora of applications in the health field and in the materials sciences alike. The resolution limit in conventional MRI is often hardware-related. For clinical MRI, for example, this limit is typically dictated by the maximum gradient strength. Intrinsic sample properties, such as chemical shift dispersion or fast relaxation, particularly in rigid samples, are further frequently-encountered constraining factors^3^.

The spatial variation of the radiofrequency (rf) field has also been used to perform imaging using nuclear spins^4^. In the most straightforward case, spatially resolved information can be obtained from a given volume of a sample by placing it within a coil region with large rf field variations. Surface coils are particularly useful in this regard as they have a well-defined rf field profile that can penetrate the surface region of a sample to yield localized spectroscopic information, with clear uses for *in vivo* applications. Classes of ‘depth pulses’^5–8^ and pulse sequences^9^ were later developed to be used in conjunction with surface coils to further enhance the spatial selectivity. These experiments form part of a larger class of MR imaging methods that can be used to study planar samples^10^.

In conductors, there is an intrinsic spatial dependence of the rf field due to induced eddy currents on the surface of the object that oppose the propagation of the wave into the medium. The rf field decays exponentially when it enters a conducting region within a characteristic length, called the skin depth,

1 $$\delta =\sqrt{\frac{1}{\pi \mu \nu \sigma }},$$

where *ν* is the frequency of the field, *μ* the permeability of the conductor and *σ* its conductivity. This effect has profound implications for the sensitivity of magnetic resonance (MR) techniques, which rely on radio frequency fields to excite and detect precessing spins from within conducting regions^11–17^.

In the following, we describe a microscopy technique that exploits the intrinsic changes imparted on the rf field when it enters a good conductor, rather than using intrinsically designed magnetic field profiles^18^ or stray magnetic field gradients^19^. Conducting systems offer unique challenges compared to those tackled by the ‘depth pulse’ and related techniques described above, due to the fast *T*_1_ and *T*_2_ relaxation of the nuclear spins and the intrinsic shape of the rf field profile. Our approach, termed Slice Microscopy in Conductors (SMC), exploits these traits and provides the ability to select slices within the objects. The achievable slice resolution with SMC is of the order of *δ*/10, which, due to the *ν* ^−1/2^ dependence in Eq. 1, allows for a range of resolutions depending on the experimental parameters. For example, *δ* = 12.3 *μ*m for nuclear spins of ^7^Li in metallic lithium at a magnetic field of 9.4 T (larmor frequency, *ν*_n_ = 155 MHz) and hence SMC can obtain slices with a resolution of approximately 1 *μ*m. For a corresponding electron spin transition, GHz frequencies would be relevant, and the skin depth would be in the range of $\delta \approx 1\,\mu {\rm{m}}$ and thus the SMC resolution would be of the order of 100 nm. The sequence can be combined with other MR sequences or imaging techniques to take depth-dependent measurements.

## Theory

The rf field entering a conducting region can be determined according to ref. 20 as

2 $${B}_{1}({\bf{r}})={B}_{10}{e}^{-\beta {\bf{n}}\cdot {\bf{r}}}{e}^{i\alpha {\bf{n}}\cdot {\bf{r}}-i\omega t},$$

with *B*_10_ the rf field at the surface of the conductor, **n** denoting the propagation direction, **r** the location vector, and *α* and *β* the real and imaginary parts of the wave vector, *κ* = *α* + *iβ*, defined by ref. 20,

3 $$\{\begin{array}{c}\alpha \\ \beta \end{array}\}=\sqrt{\mu \varepsilon }\frac{\omega }{c}{[\frac{1}{2}\sqrt{1+{(\frac{2\sigma }{\nu \varepsilon })}^{2}}\pm \frac{1}{2}]}^{\frac{1}{2}}.$$

Here, *ε* is the dielectric constant of the conductor and *c* the speed of light in a vacuum. For a good conductor $(\frac{2\sigma }{\nu \varepsilon })\gg 1$ and $\alpha \approx \beta =1/\delta$ (the inverse of the skin depth constant defined in Eq. 1), resulting in the same depth-dependence for both the phase and amplitude of the wave. As an example, for lithium metal, $\sigma =1.08\times {10}^{7}\,{\rm{S}}\,{{\rm{m}}}^{-1}$ and $\varepsilon \approx {\varepsilon }_{0}=8.85\times 1{0}^{-12}\,{\rm{F}}\,{{\rm{m}}}^{-1}$ at radio frequencies. Using these values, one can estimate that Li would be a good conductor in the frequency regime $\nu \ll 2.44\times {10}^{18}\,{\rm{Hz}}$, and thus well beyond the radio-frequency and microwave regions.

When incident on a well conducting surface, assuming that the surface extends to infinity, the boundary conditions dictate that only the rf field parallel to the surface remains, and the field within the conductor in the rotating frame can be described by

4 $${\tilde{B}}_{1}(r)={B}_{10}{e}^{-\beta r}{e}^{i\beta r},$$

where *r* denotes the penetration distance from the surface. The flip angle imparted on the spin magnetization by this field is given by

5 $${\alpha }_{{\rm{p}}}(r)=\gamma \tau |{B}_{10}|{e}^{-\beta r}={\alpha }_{{\rm{p}}}\mathrm{(0)}{e}^{-\beta r},$$

where *α*_p_(0) is the flip angle at the surface of the τconductor.

Using the principle of reciprocity, the detection of the NMR signal yields a doubling of the phase^21^. The voltage induced in the detection coil is given by the integral of the contributions from each depth,

6 $$\varepsilon =-2i\omega {M}_{0}\frac{{B}_{10}^{2}}{|{B}_{10}|}{\int }_{r=0}^{\infty }{e}^{2i\beta r}{e}^{-\beta r}\,\sin \,[{\alpha }_{{\rm{p}}}(r)]\,dr.$$

The phase term *e* ^2*iβr*^ governs the extent of constructive or destructive interference between the signals from different depths. An expression equivalent to Eq. 6 was earlier derived by Mehring *et al*.^22^.

**Figure 1:**
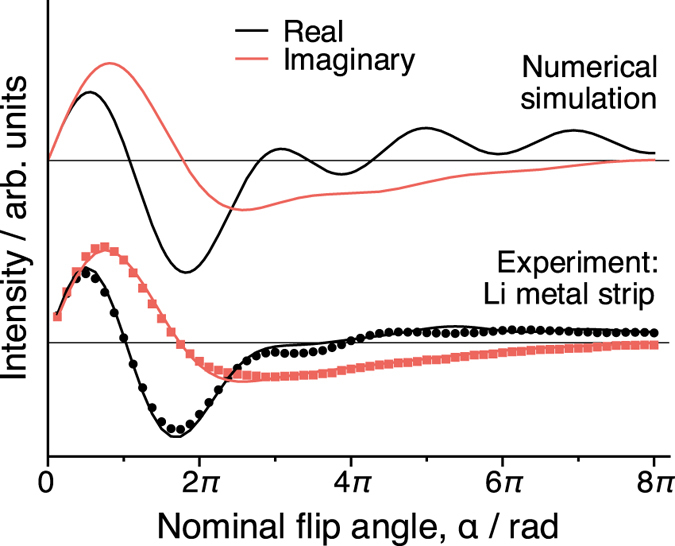
Experimental ^7^Li NMR nutation curve for a lithium metal strip plotted with the numerical solution to Eq. 6. The lines fit to the experimental data (points) were calculated from Bloch simulations (*T*_1_ = 150 ms, *T*_2_ = 600 *μ*s as measured for ^7^Li metal and 20% dispersion in *B*_10_ was assumed). The experimental flip angle is calibrated using LiCl(aq) (*τ*_*π*/2_ = 38 *μ*s).

A sensitive measure of the full form of Eq. 6 is a nutation experiment in which the MR signal is measured as a function of the flip angle, *α*_p_, which is varied experimentally by changing the *B*_1_ pulse duration, *τ*. An experimental ^7^Li NMR nutation curve performed on a rectangular piece of natural abundance lithium metal (thickness $\gg$ *δ*) is shown in Fig. 1 along with a numerical simulation of Eq. 6. There is excellent agreement between the experimental curve and the calculated one, particularly at lower flip angles <3*π*. Bloch equation simulations^23^ including relaxation during the pulse and rf inhomogeneity (20% variation in *B*_10_^13^) account for the differences for *α* > 3*π* and produce a good fit with the experimental results.

Previously, the spatial dependence of the signals was recovered by Fourier Transformation of a nutation curve^24, 25^. This approach requires relatively long acquisitions and the ability to acquire nutation curves out to long pulse durations in order to avoid truncation, which is problematic with fast relaxation.

The strategy pursued here is to preserve the magnetization at a given depth inside a conductor, while saturating it at other locations. The spatial position and thickness of the detected slice can be explicitly controlled, with each 1D NMR experiment acquiring independent slices. For samples with irregular morphologies on the order of the skin-depth, the regions selected in this fashion would become non-planar and the signal could be seen as indicative of the particle shape.

## Results and Discussion

**Figure 2:**
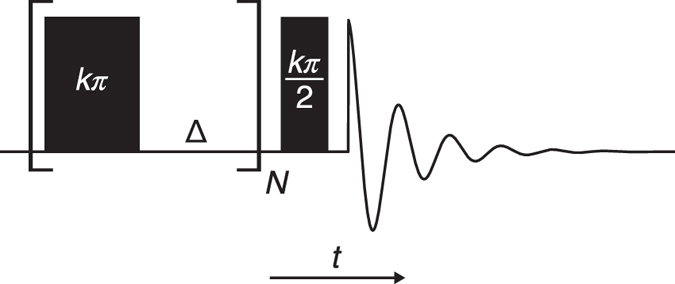
Timing diagram for the Slice Microscopy in Conductors (SMC) pulse sequence. The sequence consists of a saturating chain of *N* pulses separated by time delays Δ, followed by a readout pulse. The variable, *k*, modifies the pulses to control the depth of the selected slice, as detailed further in the main text.

Figure 2 shows the SMC pulse sequence, which consists of a chain of *N* pulses of flip angle *kπ* separated by time delays Δ, and followed by a readout *kπ*/2 pulse. All pulse flip angles are specified at the surface of the conductor, and will hence have smaller values within the conductor according to Eq. 5. For *k* > 1, there will be locations within the conductor experiencing a flip angle of *π* and the magnetization will be inverted. During the delays that follow, the transverse magnetization is allowed to decay by the *T*_2_ mechanism. In addition, one could use phase cycles to help remove this magnetization, for example, the very efficient cogwheel cycles^26^. After several cycles of this pulse-delay period, only the repeatedly inverted magnetization will persist. The final *kπ*/2 pulse reads out the stored *z*-magnetization.

**Figure 3:**
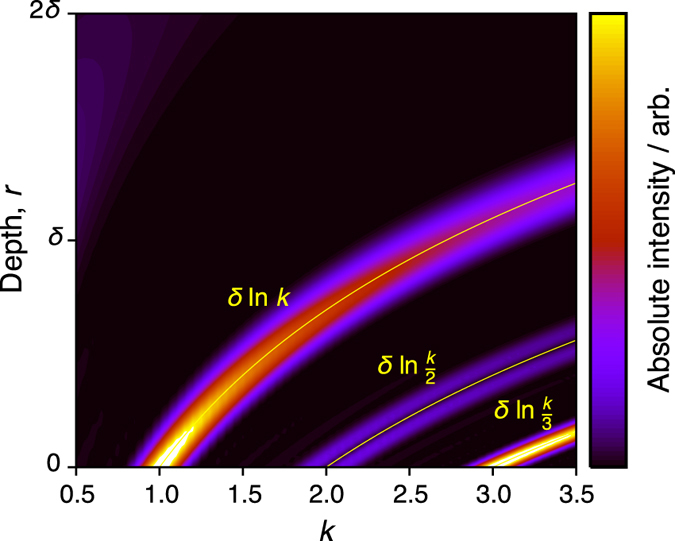
Simulation of the absolute, detected signal for different *k* at every depth in a conductor, using the SMC sequence with *N* = 16, Δ = 800 *μ*s, *T*_1_ = 170 ms, *T*_2_ = 600 *μ*s.

The effects of the SMC sequence are now considered for samples wherein both *B*_10_ and an analytical solution to *B*_1_ inside the conductor are known. For a metal film with thickness much larger than *δ* the field *B*_1_ is given by Eq. 2 and *B*_10_ is controlled by orienting the major face of the film with respect to the incoming *B*_1_ field direction^13, 27^. The behaviour of the SMC sequence for this case is simulated by solving the Bloch equations including relaxation and experimental parameters appropriate for ^7^Li metal. Figure 3 shows the results of the simulation as a function of *k* and position, *r*. When *k* < 1 there is negligible signal intensity in the region *δ* < *r* < 2*δ* where the saturation of the signal is incomplete at these low *k* values and low flip angles. This unwanted signal could be further minimized by increasing *N*. The total suppression of the conductor signal when *k* < 1 could make the SMC pulse chain useful for saturating any unwanted signal from a conductor in an MR spectrum or image.

For $k\geqslant 1$ there is an intense signal at a location *r*_sel_ in Fig. 3 where the flip angles are *π* during the saturation chain, i.e. ${\alpha }_{{\rm{p}}}({r}_{{\rm{sel}}})=\pi =k\pi {e}^{-\beta {r}_{{\rm{sel}}}}$, giving

7 $${r}_{{\rm{sel}}}=\delta \,\mathrm{ln}\,k.$$

Figure 3 shows there to be ‘overtones’ of signal intensity at *r* = *δ* ln (*k*/*n*), where $n=2,3,\ldots$. These signals occur where the flip angle is a multiple of *π*, i.e. *α*_p_(*r*) = *nπ*. The readout pulse flip angle is *α*_readout_(*r*) = *nπ*/2 at the selected positions. Therefore, odd overtones carry a substantial intensity, while for even overtones, signals experiencing a readout pulse slightly larger and smaller than *π* cancel each other, as indicated by the simulation results. A composite readout pulse with an appropriate phase cycle could be used to suppress these overtones. One simple example would be a $\{\tfrac{k\pi }{2}(x);\tfrac{k\pi }{6}(x,-x)\}$ composite pulse with a 2 step phase cycle on the second pulse component and the receiver. This procedure would cancel the signal from the *n* = 2 and 3 components but also scale the *n* = 1 component to 87% of its full amplitude.

**Figure 4:**
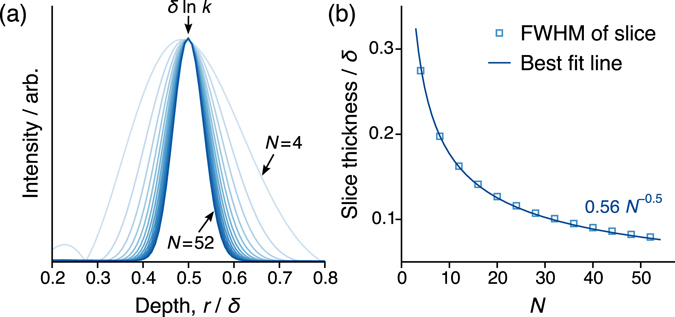
(**a**) Simulated slice profiles using SMC with *N* = 4 to 52 (in steps of 4), using a single value of *k* = 1.65. The maximum value of the curves is normalized to allow for a direct comparison of the slice width. (**b**) Calculated full width at half maximum (FWHM) of the lineshapes in (**a**) plotted against *N*. The Bloch simulations were performed with Δ = 800 *μ*s, *T*_1_ = 170 ms, *T*_2_ = 600 *μ*s and *τ*(*π*-pulse) = 10 *μ*s.

Experimentally, the application of the SMC sequence using a given value of *k* will result in an NMR signal originating from a discrete spatial region in a conductor; a slice. The simulated behaviour of this slice profile for *k* = 1.65 with varying *N* is shown in Fig. 4a, where it is demonstrated that the choice of *N* controls the thickness of the slice profile. The larger *N*, the narrower the selected slice. As shown in Fig. 4b, the relationship between the full width at half maximum (FWHM) of the slice profile and the value of *N* is highly predictable, fitting very well to a ${w}_{0}/\sqrt{N}$ dependence. Here, *w*_0_ is a fitted parameter that is associated with the intrinsic dispersion of spins selected by each pulse and their *T*_2_ constants. The lineshapes in Fig. 4a are very close to Gaussian, although there is a notable distortion in the profile when *N* = 4 arising from the *e* ^−*βr*^ dependence of the detection field that skews the lineshape towards low *r* values.

**Figure 5:**
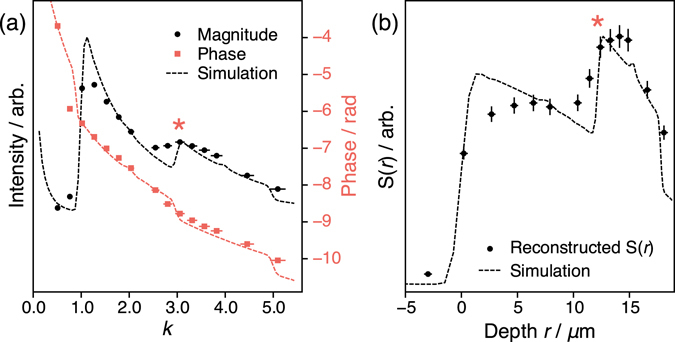
^7^Li experimental results obtained on a lithium metal strip. (**a**) Raw magnitude and phase data obtained by varying *k* (by changing *τ*) in the pulse sequence in Fig. 2 with *N* = 16, Δ = 800 *μ*s and *τ*(*π*-pulse) = 16 *μ*s for *k* = 1, as calibrated on 1 M LiCl(aq). The intensity of the on-resonance position in the spectrum is plotted. The simulated data are taken from the sum over all depths in Fig. 3. (**b**) Reconstructed *S*(*r*) slice profile from the results shown in (**a**). Error bars are derived from the errors in the pulse calibration (0.25 *μ*s error assumed on the calibrated *π*/2 pulse) and NMR signal intensities (taken as the standard deviation of the noise in each spectrum). Where errors are not visible they fall within the marker size. The asterisks indicate a jump due to the additional overtone signal at *k* = 3 and $r=\delta \,\mathrm{ln}\,3\approx 12\,\mu {\rm{m}}$.

Experimental results using SMC (Fig. 2) on a lithium metal strip are shown in Fig. 5(a) with the corresponding simulation results. The experimental results show a good fit to the simulated curve. According to Fig. 4 the slices can be expected to be 2–3 *μ*m thick.

It is possible to convert the *k* axis to a depth axis using Eq. 7. The *y* axis, Sig(k) can also be converted into a profile of the spin density as a function of depth, *S*(*r*) by accounting for the exponential dependence of the detection field,

8 $${\rm{Sig}}({\rm{k}})=S(r){e}^{-\beta {r}_{{\rm{sel}}}},$$

9 $$S(r)=k\,\mathrm{ln}\,[\text{Sig}({\rm{k}})],$$

using Eq. 7 to substitute the experimental parameter *k* for *r*_sel_. By transforming the axes in this manner, a depth profile through the surface of the object is obtained. For the lithium metal strip, this profile (Fig. 5(b)) is flat in the region 0 < *r* < *δ* as expected for a uniform metal. For *δ* < *r* the profile represents the sum of the *n* = 1 and the *n* = 3 bands. This contribution could be removed by the use of supercycles or specific readout pulses as mentioned above. Nevertheless, the profile in Fig. 5(b) represents a 1D slice of the metal profile with micron–scale resolution in the region 0 < *r* < *δ*.

A limitation of SMC is that the simulations and the conversion to the depth-sensitive spin distribution, *S*(*r*) require knowledge of *B*_1_ and *B*_10_. Although these could be calibrated, when particle sizes and surface curvature are on the order of *δ*, further complications arise and *B*_1_ is not known analytically. The orientation of the particle surfaces with respect to the incoming rf field direction is also important. *B*_10_ will therefore be inhomogeneously distributed in powdered samples.

**Figure 6:**
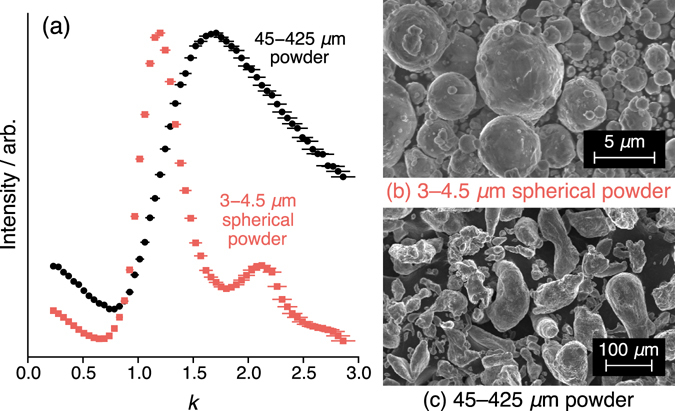
Experimental results obtained on powdered aluminium samples by (**a**) varying *k* (using a fixed pulse width, *τ*_*π*/2_ = 11 *μ*s and changing the pulse power, with the *k* = 1 pulse power calibrated with a 1 M Al(NO_3_)_3_ solution) in the pulse sequence shown in Fig. 2 with *N* = 8 and Δ = 80 *μ*s. The intensities of the on-resonance positions in the spectra are plotted. Error bars are calculated following the methodology described in the caption to Fig. 5. Where errors are not visible, they fall within the marker size. (**b**,**c**) SEM images of the two samples. The second peak at higher *k*-value for the smaller particles is likely a consequence of the *n* = 2 overtone signal, which is due to the fact that the particle size is <*δ*/4. As a result, signals from regions experiencing a readout pulse slightly larger and smaller than *π* do not cancel each other completely.

Despite the difficulties in evaluating the exact rf field in powdered samples, SMC can be useful in determining the approximate particle size and in distinguishing between rf field distributions (Fig. 6). Moreover, by choosing appropriate values of *k*, the SMC pulse chain can be used to selectively excite the MR signal from some particles while saturating others. For example, for the two samples shown in Fig. 6, an SMC excitation at *k* = 1.8 selectively excites the larger particle distribution and saturates magnetization elsewhere, thus allowing the relative populations and information about the particle size distribution to be ascertained. In particular, if the particle size is on the order of the skin-depth, these particles can be easily saturated by using a large *k*. By contrast, using a small *k* will favor the smaller particles. In the results shown in Fig. 6, the peak close to *k* = 1 indicates particle sizes on the order of the skin-depth, while there is a broad distribution for the other sample, with significant signal up until *k* = 3.

While there exist many techniques for particle-size measurements, the NMR-based approach has the advantage of being compatible with analyzing opaque objects. Clearly, a limitation of the technique is that high spatial localization is only given along one dimension. When analyzing surfaces with uniform conductivity values, the SMC technique would, however, allow, for example, the measurement of the propagation of different metals into a bulk conducting region as a function of time or a particular driving force. One specific example would be the study of ^6^Li and ^7^Li isotope ratios at different depths of a lithium metal electrode, when supplied with an excess of one isotope at the initial point^12^. The main constraint in such an application (aside from sensitivity constraints) is simply given by the strength of the rf field: The rf power needs to be significantly stronger than 1/*T*_2_, while *T*_1_ needs to be significantly longer than the duration of the pulse train.

## Conclusion

The spatial dependence of the rf field within a conductor makes it useful for the selection of slices within the material and to produce depth-selective measurements with NMR and MRI. Using a pulse sequence based on repeated inversion and saturation steps, slices with sub-micron thickness can be selected at a well defined depth from the surface of a conductor. The slice position and width can be controlled by pulse sequence parameters. In systems where the rf field profile of the conductor is not known analytically or when inhomogeneities in the sample shape and surface orientation result in a distribution of rf field intensities at the conductor surface, the method can be used to discriminate between different particle distributions, to selectively excite a signal from some particles and not from others, or to provide a fast measure of surface area and morphology.

The pulse sequence can also be used to prepare the magnetization prior to the application of a more advanced readout sequence, allowing the depth dependence of other properties to be explored. This could include, for example, imaging sequences, cross polarization sequences to transfer the magnetization to secondary nuclei (also allowing distance measurements) and *T*_2_ or *T*_1*ρ*_ relaxation measurements. Applying SMC in these ways could provide new routes to understanding the properties of conducting surfaces at sub- micron length scales. These capabilities may make the technique suitable for applications in studies of lithium or sodium battery systems, superconductors or electronics components. Further applications could include metallurgy, when location-specific composition is probed, or where probes of metal diffusion would be desired.

## Methods

The ^27^Al SMC results and the nutation curve for ^7^Li metal were obtained on a Bruker Ultrashield 9.4 T Avance I spectrometer operating at 155.5 MHz for ^7^Li and 104.3 MHz for ^27^Al. The ^7^Li nutation curve was acquired using a Bruker ^1^H^27^Li WB40 birdcage coil on a strip of natural abundance lithium metal (Aldrich 99.9%) cut to ca. 0.4 × 8 × 15 mm and sealed inside a 10 mm NMR tube. The aluminium SMC experiments were acquired on powdered aluminium samples with small (Alfa Aesar, APS 3.0–4.5 micron, 97.5% metals basis) and large (Alfa Aesar, −40 + 325 mesh, 99.8% metals basis) particle sizes, using a 4 mm solid-state HX Bruker probe (chosen for its strong *B*_1_ field, samples were not spun). The aluminium powders were diluted 1:1 by weight with CaCl_2_ in order to ensure a more uniform *B*_1_ field. A Bruker Avance-500 NMR spectrometer with a BBO probe tuned to ^7^Li at 194.4 MHz was used to collect data for the SMC sequence on a natural abundance lithium metal strip (Aldrich 99.9%) cut to ca. 0.4 × 3 × 6 mm and sealed inside a 5 mm NMR tube. In all cases spectra were acquired on resonance with the center of the metal peak and the plotted intensity profiles correspond to the on-resonance position in the spectrum.

SEM images of the powdered aluminium samples was obtain using a MERLIN (Carl Zeiss) field emission scanning electron microscope (FESEM) with an SE2 (Everhart-Thornley type) detector.
